# Effectiveness and acceptability of biometrics to evaluate intervention coverage and contamination in a cluster randomised trial of community-based sexual and reproductive health services for youth in Zimbabwe

**DOI:** 10.1136/bmjopen-2025-107583

**Published:** 2026-07-15

**Authors:** Victoria Simms, Tsitsi Bandason, Ethel Dauya, Chido Dziva Chikwari, Chris Grundy, Mandikudza Tembo, Constancia Mavodza, Owen Mugurungi, Tsitsi Apollo, Riffat Ashrafee, Demetris Demetriou, Anindya Sharma, Richard J Hayes, Katharina Kranzer, Rashida A Ferrand

**Affiliations:** 1 MRC International Statistics and Epidemiology Group, Department of Infectious Disease Epidemiology, London School of Hygiene and Tropical Medicine, London, UK; 2 The Health Research Unit Zimbabwe, Biomedical Research and Training Institute, Harare, Harare Province, Zimbabwe; 3 Department of Global Health and Development, Faculty of Public Health and Policy, London School of Hygiene and Tropical Medicine, London, UK; 4 AIDS and TB Unit, Ministry of Health and Child Care, Harare, Zimbabwe; 5 Simprints, Cambridge, UK; 6 Department of Clinical Research, London School of Hygiene and Tropical Medicine, London, UK; 7 Institute of Infectious Diseases and Tropical Medicine, LMU University Hospital, Munich, Germany

**Keywords:** Africa South of the Sahara, EPIDEMIOLOGY, BIOTECHNOLOGY & BIOINFORMATICS

## Abstract

**Objectives:**

Low intervention uptake and contamination can dilute effects of cluster randomised trials (CRTs). We investigated the feasibility of digital fingerprints to assess intervention coverage and contamination in a CRT of community-based sexual and reproductive health services for youth (CHIEDZA).

**Methods:**

24 clusters in Zimbabwe were randomly allocated to intervention/control. In intervention clusters, CHIEDZA services were provided in community halls for 30 months. A population-based survey of youth aged 18–24 (700/cluster) was conducted to ascertain impact. Digital fingerprints were collected from service attendees and survey participants, and the datasets were linked to assess intervention coverage at population level in intervention clusters and contamination in control clusters. Multilevel logistic regression estimated the association of hall distance with service uptake.

**Results:**

Between April 2019 and March 2022, 36 991 clients attended the CHIEDZA service and 99.9% used biometric registration. In the survey 13 675/17 682 (77.3%) participants completed biometric registration: 1182 refused, 1235 bypassed and 1590 could not register.

CHIEDZA service coverage in the intervention clusters was 23.1% and contamination was 3.7%. Against biometric registration match, self-reported service attendance had 75.3% sensitivity (95% CI 73.1% to 77.5%) and 92.7% specificity (95% CI 92.0% to 93.4%). Odds of CHIEDZA service use reduced by 52% for every 1km distance (OR: 0.48 95% CI 0.44 to 0.54).

**Discussion:**

Biometric identification was feasible and acceptable in a community setting without time pressure. In population-based surveys additional technological challenges emerged. Biometrics enabled good estimation of intervention coverage and validated self-reported data. Youth community services must overcome distance barriers.

**Conclusion:**

Biometric identification is useful for assessment of CRT coverage and contamination.

**Trial registration number:**

https://clinicaltrials.gov/study/NCT03719521.

**STRENGTHS AND LIMITATIONS OF THIS STUDY**

- The study recruited a large, population-representative sample of 17 682 youth in urban and semi-urban Zimbabwe.
- A validation dataset was used to determine accuracy of matching fingerprints.
- A small number of data collectors systematically bypassed biometric registration in the last two months of survey data collection.
- Reasons for refusal of biometric registration were not recorded.

## Background

 Digital biometrics are increasingly used for identification and monitoring of individuals in targeted public health interventions.^1^ Biometrics have potential for use among clients who do not wish to leave identifying information, and may be especially useful where individuals might fear discrimination or have inadequate documentation.^1^ Digital fingerprints have been used in clinical settings, for example, a group of 300 people living with HIV in Kenya^2^ and for antenatal care in Malawi,^3^ and in rural Ghana to link hospital and community records.^4^ However, marginalised groups have expressed concerns about biometric registration, such as infringement of privacy and exposure to risk of legal action.^5 6^

Cluster-randomised trials (CRTs) of public health interventions are a possible application of this technology. CRTs are particularly useful for evaluating interventions that can only be delivered at group level, and facilitate assessment of population-level efficacy.^7^ Trial efficacy is driven not just by inherent effectiveness of an intervention but also its coverage. Contamination between intervention and control clusters can also undermine the efficacy estimated by a CRT. Intervention uptake is usually assessed by self-report which is vulnerable to bias. Digital biometrics offer an innovative and more objective method, but its feasibility and acceptability is unknown. In Uganda, technological problems impeded implementation of digital fingerprinting in a tuberculosis contact tracing study.^8^

We aimed to investigate the feasibility, accuracy and applicability of digital fingerprints in a CRT of community-based HIV and sexual and reproductive health (SRH) services for youth in Zimbabwe. We hypothesised that delivery of integrated, youth-friendly services outside a health facility setting would increase access, acceptability and uptake and thus have population-level impact on health outcomes, which were assessed through a population-based survey.^9^ We used digital fingerprints to register clients accessing the service on each visit (intervention), and also in the outcome survey, enabling dataset linkage. As well as assessing feasibility and accuracy of the technology, we estimated SRH service uptake at population level in the intervention clusters (coverage) and in the control clusters (contamination), and investigated factors associated with uptake.

## Methods

### Study design and setting

The CHIEDZA clusters were 24 demarcated areas containing a community hall and primary care clinic with a population of 2500–4500 youth, randomised 1:1 to the trial intervention (community-based integrated HIV and SRH service for 30 months) or to control (existing, mainly clinical-based, services).^10^ All cluster residents aged 16–24 years were eligible to attend and receive services, including HIV testing and management, STI screening and treatment, contraception, menstrual health management and counselling. The service was operated from community halls equipped with entertainment facilities to create a welcoming environment, and small booth tents for privacy. Community mobilisers sensitised cluster residents about the service.

### Biometric data collection and definition of cut-points

After consenting, CHIEDZA service clients completed biometric registration with prints from four fingers (left and right thumb and index) using a fingerprint scanner, with an alternative option for manual registration using a paper-based form. Clients could make repeat visits, and fingerprint identification was used to determine the date of registration and services previously received. Data were collected using Samsung Galaxy A7 V.10.4 (2020) Android tablets, Bluetooth linked to the Simprints Vero fingerprint scanner with SurveyCTO for data collection and Simprints ID software to store the biometric data.^1^ All fingerprint registrations were converted into a 32-character Globally Unique Identifier (GUID) stored in a SurveyCTO database. Actual fingerprint data were stored on the Simprints server and data were encrypted at every stage of data collection.

Accuracy of the digital fingerprint system was calculated with a small dataset of 300 service clients, each scanned two times. The biometric profiles of each pair of scans were compared with create an aggregated comparison score which was the mean comparison score of each of the four fingers. The false acceptance rate (FAR) and false rejection rate (FRR) were calculated over a range of comparison score cutpoints to identify the optimal cutpoint for further analysis.

## Population-based survey

The CRT primary outcome was prevalence of unsuppressed HIV viral load among youth living with HIV, evaluated using a cross-sectional population-based survey of 700 youth aged 18–24 years per cluster. Survey participants were registered using Simprints in the same way as service clients. Survey participants were asked whether they had ever heard of CHIEDZA or accessed its services. Other variables collected included length of residence at current address (<12 months, 12–24 months and >24 months), age, sex, marital status (never married, married or living together, divorced/widowed/separated), education level (none, primary, secondary, tertiary), current main activity (none, in school, registered business/formal job, informal sector job) and whether ever had sexual intercourse.

For the survey, each cluster was mapped using OpenStreetMap and divided into road sections measuring c.100-300m, except one cluster with limited road infrastructure which was divided into zones. The size of sections was based on population distribution from the Zimbabwe 2012 Census. A sample of road sections was randomly selected within each cluster and all households in these sections were enumerated to identify residents aged 18–24 years. If the sample was inadequate to recruit 700 youths, a further sample of road sections was taken and visited sequentially to maintain randomness until 700 was reached. A median 100 sections (IQR 88–148) were surveyed per cluster. All those eligible were contacted, if possible, and asked to participate in the survey and provide fingerprint registration.^10^ The coordinates of each dwelling were collected using the tablet’s in-built Global Positioning System (GPS).

Initially, data collectors were unable to easily bypass fingerprint registration because all processes, including bypass and refusals, were required to be processed through the Simprints ID application. On 21 April 2022 the bypass process was made easier in response to staff requests, due to technical challenges.

## Data analysis

Analysis was conducted using Stata V.18.0 and R V.4.3.3. The dataset of service clients was linked to the survey dataset by fingerprint ID, within province. If a service client fingerprint was linked to >1 survey participant, these records were excluded. Feasibility was assessed from the proportion of survey participants who gave a digital fingerprint, the proportion of occasions when data collectors bypassed the process, accuracy against self-reported attendance and extent of duplicates. Univariable mixed-effects logistic regression with a random term for cluster was used to investigate whether demographic characteristics were associated with refusal of biometric registration. Intervention coverage was defined as the proportion of fingerprinted survey participants in the intervention clusters who matched the service client dataset. Contamination was defined as the proportion of fingerprinted survey participants in the control clusters who matched the service client dataset (online supplemental figure S1).

Distance from each survey participant’s residence to the community hall was calculated, both as Euclidean (straight line) distance, using the Stata vincenty module, and walking distance, using the Google Apps script DirectionFinder based on road infrastructure recorded in Google Maps. Multilevel mixed-effects logistic regression was used to examine the association of service utilisation with walking distance and other covariates, accounting for cluster. Socioeconomic status was determined from asset ownership variables using factor analysis and divided into quintiles.

### Patient and public involvement

The CHIEDZA service intervention was co-designed in collaboration with youth in two participatory workshops in Zimbabwe.^11^ Digital biometrics were incorporated in the study design following discussion with youth. An information film about the survey was designed in collaboration with youth. Results were disseminated to participant communities in collaboration with the youth advisory group.

## Results

### Feasibility and accuracy

**Figure 1:**
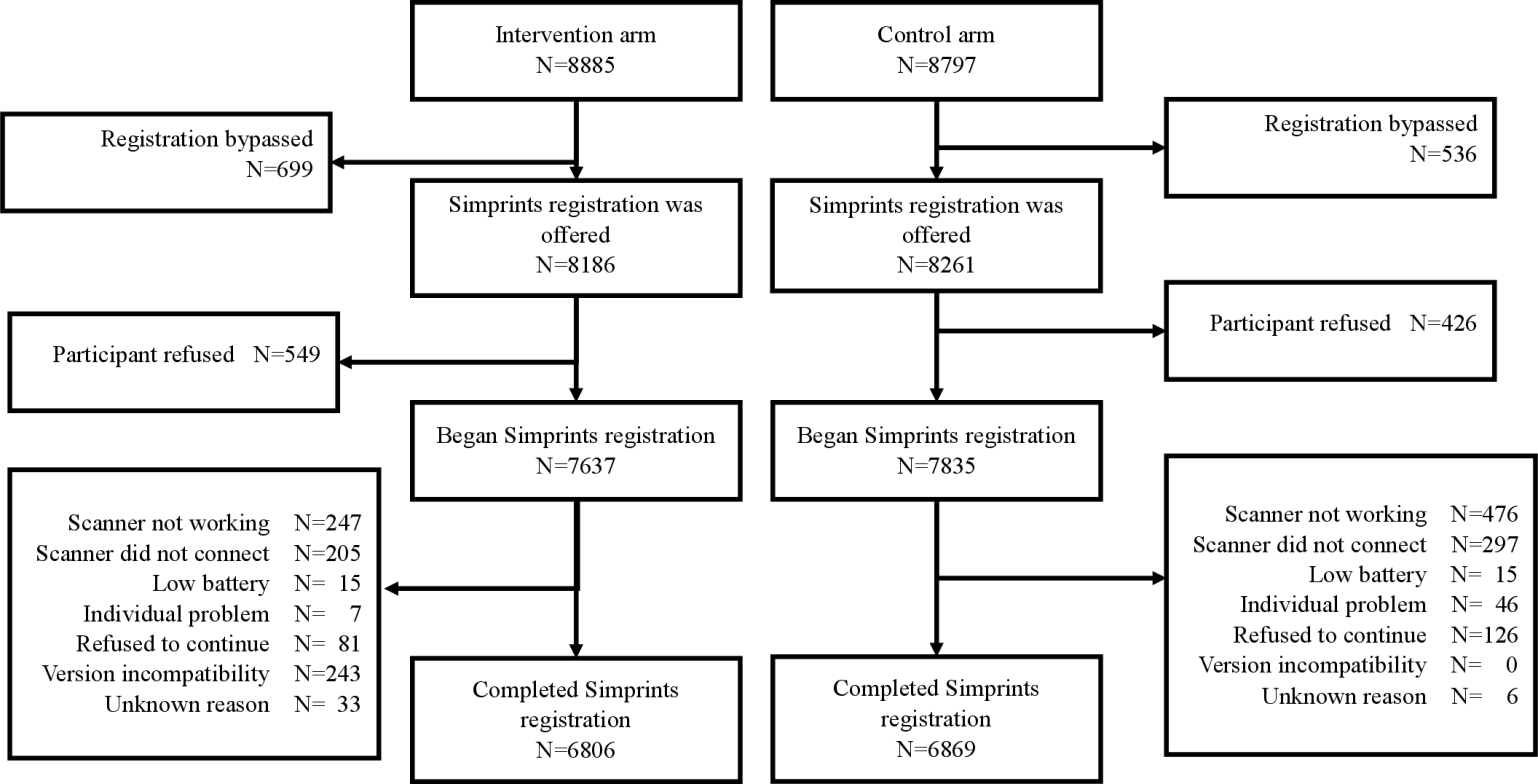
Survey enrolment flowchart.

From 1 April 2019 to 31 March 2022, 36 991 unique clients attended the CHIEDZA service in the 12 intervention clusters, of whom 36 957 (99.9%) registered their fingerprints. 95 clients (63 in Bulawayo, 17 in Mashonaland East and 15 in Harare) attended >1 CHIEDZA community centre. The survey took place from 4 October to 15 December 2021 in Harare, 4 January to 5 March 2022 in Bulawayo and 4 April to 2 June 2022 in Mashonaland East, beginning within 1 month of service closure. 90 staff conducted data collection (45 in Harare/Mashonaland East and 45 in Bulawayo), with median enrolment of three participants/day (IQR 3–4, range 1–10). In total 17 682 participants were enrolled of whom 13 675 (77.3%) successfully registered their fingerprint (figure 1). Reasons for non-registration were that the researcher bypassed the process (N=1235), the participant refused to give fingerprints (N=975) or the registration process was begun but not completed (N=1797). Of those who started the process but did not complete it, 207 refused (95 for religious reasons, 66 because of data concerns and 46 gave no reason), for a total of 1182 refusals (975+207).

Apart from refusal, the other causes of non-completion of biometric registration were: the scanner was not working (N=723), did not connect to the tablet (N=502) or had low battery (N=30); software version incompatibility (N=243); individual reasons which prevented fingerprint detection (eg, the participant had grease on their hands) (N=53); and 39 were for unknown reason. The version incompatibility problem occurred during the first 3 weeks of data collection in Harare (online supplemental table S1). Automatic software updates were disabled on the tablets, causing compatibility issues between newer scanners and the old software. The app recorded the event as ‘register biometrics complete’ although a biometric ID was not stored, so data collection staff did not realise that registration had failed. This problem was resolved by the data office on 23 October 2021 and did not re-occur.

Until 20 April 2022 only 149 (1.1%) participants bypassed registration out of 13 367 recruited. From 21 April, when bypass was made easier, until the survey ended on 2 June 2022, 1086/4315 (25.2%) participants bypassed fingerprint registration. Each data collector bypassed a median 7.4% of registrations (IQR 0–35%) during this time, and four data collectors bypassed >90%.

Excluding bypasses, 667/6334 (10.5%) men refused versus 515/10,112 (5.1%) women, with no difference by age (table 1). Refusal among eligible participants was 9.9% in Bulawayo and Mashonaland East but 2.3% in Harare, and ranged by cluster from 0.3% to 27.5% (median 6.7%, IQR 2.3–10.0%). In the intervention clusters, fingerprint refusal was 3.8% among participants who self-reported accessing CHIEDZA services versus 8.9% among participants who said they had not.

**Table 1:** Survey biometric registration refusal by socio-demographic and trial characteristics, among those who did not bypass

Variable	Category	Offered biometric registration	Refused biometric registration	OR (95% CI)	P value
	N	16 447	1182		
Sex (missing for 1)	Male	6334	667 (10.5%)	1	
	Female	10 112	515 (5.1%)	0.54 (0.48 to 0.62)	<0.001
Age	18–20	8588	606 (7.1%)	1	
	21–25	7859	576 (7.3%)	1.11 (0.98 to 1.25)	0.11
Province	Harare	5794	132 (2.3%)	1	
	Bulawayo	5911	582 (9.9%)	4.96 (2.26 to 10.90)	<0.001
	Mashonaland East	4742	468 (9.9%)	6.32 (2.88 to 13.86)	
Trial arm	Intervention	8186	630 (7.7%)		
	Control	8261	552 (6.7%)	1.16 (1.03 to 1.31)	0.012
Self-report accessing CHIEDZA service (intervention arm)	Yes	1964	74 (3.8%)		
	No	6222	556 (8.9%)		
Education level	Did not complete primary	109	15 (13.8%)	0.93 (0.59 to 1.46)	<0.001
	Completed primary	808	46 (5.7%)	0.59 (0.45 to 0.78)	
	Completed form 4	2851	284 (10.0%)	0.79 (0.63 to 0.99)	
	Completed form 6	587	68 (11.6%)	0.92 (0.71 to 1.19)	
	Post-secondary	387	55 (14.2%)	1	
Socioeconomic status	Poorest	3647	287 (7.9%)	1	0.25
	2	2927	196 (6.6%)	0.85 (0.69 to 1.03)	
	3	3322	235 (7.1%)	0.90 (0.74 to 1.09)	
	4	3237	208 (6.4%)	0.82 (0.67 to 1.01)	
	Least poor	3317	259 (7.8%)	0.96 (0.79 to 1.17)	

In the test dataset the optimal cutpoint for comparison score was determined to be 21.5, with FAR of 0.01% and FRR of 0.94% (online supplemental figure S2). At this cutpoint, 128 service clients appeared to match >1 survey participant. These records were excluded from the dataset of 36 991 clients, leaving 36 863. Out of 1826 survey participants who linked to the service dataset, 10 participant fingerprints (0.5%) linked to >1 client (in 2 cases to 3 clients, in 8 cases to 2 clients). On checking, these client pairs usually shared a birthdate and were at the same cluster, and on one occasion were seen on the same day. The most likely explanation is that these were visits by the same client, who was not recognised as a match during the biometric registration and was re-registered. In these cases, one record from each group was retained to link with the survey participant.

### Intervention coverage

Using a cutpoint of 21.5, coverage in the intervention arm was 23.1% (95% CI 22.1% to 24.1%) (table 2). Uptake was higher in women than men and higher in Bulawayo than the other provinces. Overall, 10.5% of matches in the intervention arm were cross-cluster; the survey participant attended CHIEDZA services not in their cluster of residence, but in another clusters within the province. Participants who had lived at their current address for a shorter time were less likely to have attended the CHIEDZA service, and those who had previously lived in another area or city were more likely to have attended in another cluster (online supplemental figure S3).

**Table 2:** Service uptake among prevalence survey participants who completed biometric registration (N=13 675); using cutpoint 21.5)

		N	Intervention clusters	N	Control clusters
Total		6806	1574 (23.1%)	6869	252 (3.7%)
Sex	Male	2403	460 (19.1%)	2625	54 (2.1%)
	Female	4403	1114 (25.3%)	4243	198 (4.7%)
Age	18–20	3579	852 (23.8%)	3575	125 (3.5%)
	21–24	3227	722 (22.4%)	3294	127 (3.9%)
Province	Harare	2592	471 (18.2%)	2350	73 (3.1%)
	Bulawayo	2390	790 (33.1%)	2481	122 (4.9%)
	Mash East	1824	313 (17.2%)	2038	57 (2.8%)
Self-reported accessing CHIEDZA service	No	5238	388 (7.4%)	6822	224 (3.3%)
	Yes	1568	1186 (75.6%)	47	28 (59.6%)

Among intervention arm survey participants with biometric registration, self-report against fingerprint match had positive predictive value of 75.6% (95% CI 73.4% to 77.7%), negative predictive value of 92.6% (95% CI 91.8% to 93.3%), sensitivity of 75.3% (95% CI 73.1% to 77.5%) and specificity of 92.7% (95% CI 92.0% to 93.4%).

### Contamination

At a cutpoint of 21.5, uptake in the control clusters was 3.7% (95% CI 3.2% to 4.1%). As with the intervention clusters, uptake was higher in women and in Bulawayo. In the control arm 47 participants self-reported accessing CHIEDZA services, of whom 28 (59.6%) had a fingerprint match.

Among the 1574 survey participants in the intervention clusters with a fingerprint match to the dataset of CHIEDZA service attendees at a comparison score >21.5, the median comparison score was 55.7 (IQR 41.1–70.5, range 21.5–142.7). Among the corresponding 252 control arm participants with a comparison score >21.5, the median score was 22.2 (IQR 21.9–24.1, range 21.5–122.8) Sensitivity analysis results are shown in online supplemental table S2. At a cutpoint of 23, uptake was 21.3% in the intervention clusters (coverage) and 1.3% in the control clusters (contamination). Raising the cutpoint to 23 compared with 21.5 resulted in a 7.8% reduction in the number of service clients in the intervention arm and a 65% reduction in the number of service clients in the control arm.

### Factors associated with service uptake

In the intervention clusters 8885 participants were enrolled. Five did not have an accurate GPS reading and 2805 did not have fingerprint data, leaving 6805 (table 3). A higher proportion of females (25.3%) than males (19.1%) accessed CHIEDZA services. Univariable multilevel mixed-effects logistic regression showed that utilisation of CHIEDZA services was associated with being female (OR 1.55, 95% CI 1.37 to 1.76) and with being married or cohabiting (OR 1.21, 95% CI 1.21 to 1.26) versus never married (table 3).

**Table 3:** Utilisation of CHIEDZA services in intervention arm by socio-demographic characteristics

			Used CHIEDZA service		Univariable crude OR adjusted for clustering (95% CI)	P value
Characteristic		All	No, n (%)	Yes, n (%)		
		6805	n=5233 (76.9)	n=1572 (23.1)		
Euclidean distance from home to CHIEDZA location (km)*	Mean (SD)		1.01 (0.48)	0.84 (0.31)	0.52 (0.46 to 0.59)	<0.0001
Walking distance from home to CHIEDZA location (km)*	Mean (SD)		1.42 (0.55)	1.17 (0.39)	0.48 (0.44 to 0.54)	<0.0001
Duration of residence at current address	<12 months	1728 (25.4)		208 (12.0)	1	<0.0001
	12–24 months	701 (10.3)		133 (19.0)	1.74 (1.37 to 2.22)	
	>2 years	4376 (64.3)		1231 (28.1)	2.57 (2.18 to 3.03)	
Sex	Male	2402 (35.3)		458 (19.1)	1	<0.0001
	Female	4403 (64.7)		1114 (25.3)	1.55 (1.37 to 1.76)	
Marital status	Never married	5037 (74.0)		1173 (23.3)	1	0.039
	Married/living together	1479 (21.7)		335 (22.7)	1.21 (1.04 to 1.40)	
	Divorced/widowed/separated	289 (4.3)		64 (22.1)	1.13 (0.85 to 1.52)	
Current main activity	None	3343 (49.1)		790 (23.6)	1	0.625
	Education	1927 (28.3)		446 (23.1)	0.93 (0.81 to 1.06)	
	Registered business/formal sector work	312 (4.6)		83 (26.6)	1.04 (0.80 to 1.37)	
	Informal sector work	1223 (18.0)		253 (20.7)	0.94 (0.80 to 1.11)	
Level of education	None, any primary or completed primary	365 (5.4)		75 (20.6)	1	0.0006
	Any secondary or completed secondary	5892 (86.6)		1402 (23.8)	1.19 (0.91 to 1.55)	
	Any post-secondary	548 (8.0)		95 (17.3)	0.76 (0.54 to 1.08)	
Ever had sexual intercourse	No	2287 (35.1)		476 (19.9)	1	0.0002
	Yes	4379 (64.3)		1082 (24.7)	1.28 (1.13 to 1.45)	
	Do not want to say	39 (0.6)		14 (35.9)	1.96 (0.99 to 3.90)	
Age in years	18–20	3579 (52.6)		850 (54.1)	1	0.309
	21–24	3226 (47.4)		722 (45.9)	0.94 (0.84 to 1.06)	

* Increased odds per increased kilometre of distance.

For every km increase in walking distance, the odds of service utilisation reduced by 52% (OR 0.48, 95% CI 0.44 to 0.54) and a similar margin of effect was observed using Euclidean distance (OR 0.52, 95% CI 0.46 to 0.59). Mean walking distance of service attendees was on average 0.25 km shorter than non-attendees in the same cluster, and cluster-adjusted Euclidean distance was 0.17 km shorter (online supplemental figure S2).

In multivariable sex-stratified multilevel mixed effects logistic regression models, CHIEDZA service utilisation was associated with longer duration of residence both for males and females after adjusting for sexual debut, education and distance (table 4). In males only, sexual debut was associated with increased odds of CHIEDZA service utilisation (adjusted OR (AOR) 2.01, 95% CI 1.58 to 2.57), with evidence of interaction by sex (p<0.001). Females who had post-secondary education were less likely to have used CHIEDZA services (AOR 0.56, 95% CI 0.37 to 0.86) compared with females with primary level of education, but there was no evidence of interaction by sex.

**Table 4:** Association of utilisation of CHIEDZA service with walking distance and length of residence, for outcome survey male and female participants in the intervention arm

		Males		Female	
Characteristic		Adjusted OR (95% CI)	P value	Adjusted OR (95% CI)	P value
Walking distance (km)		0.48 (0.40 to 0.58)	<0.0001	0.49 (0.43 to 0.55)	<0.0001
Ever had sexual intercourse	No	1	<0.0001	1	0.401
	Yes	2.01 (1.58 to 2.57)		1.15 (0.99 to 1.35)	
	Do not want to say	3.14 (1.10 to 8.96)		1.71 (0.64 to 4.54)	
Level of education	None, any primary or completed primary	1	0.0686	1	0.007
	Any secondary or completed secondary	1.69 (0.93 to 3.09)		1.01 (0.74 to 1.39)	
	Any post-secondary	1.08 (0.53 to 2.18)		0.56 (0.37 to 0.86)	
Duration of residence at current address	<12 months	1	<0.0001	1	<0.0001
	12–24 months	1.89 (1.03 to 3.49)		1.71 (1.30 to 2.25)	
	>2 years	3.10 (2.05 to 4.69)		2.99 (2.48 to 3.61)	

## Discussion

Our study showed that use of digital fingerprints was feasible and accurate, and these biometrics can be applied to understand intervention coverage and contamination, two aspects that critically affect the measured efficacy of interventions. Digital fingerprints had <0.1% refusal in the CHIEDA service, but we observed higher refusal among survey participants. Notably there was generally good agreement of biometrics with self-reported attendance.

There are several reasons why uptake of biometric registration was higher at the CHIEDZA services than during the prevalence survey. Each community centre had several tablets and scanners, so a non-functional piece of equipment could quickly be exchanged for a working one. Attendees may have been motivated to use biometric registration in order to access services. Manual registration was possible but was considered a tedious and time-consuming process, so staff preferred to use biometric registration whenever possible. Handwashing facilities were also readily available at community halls. By contrast, in the cross-sectional survey, there were operational challenges. The two-person mobile teams had no backup equipment with them, and were under time pressure to complete the survey. Scanner and tablet had to be reconnected frequently. New equipment was purchased as required but was not always immediately compatible with the older equipment. Survey participants had no particular motivation to consent to biometric registration. The manual registration process was deliberately made easier, with the result that staff were more likely to opt for it when they experienced or anticipated technical challenges.

Close observation of the data in real time is essential to quickly identify and resolve problems. This was particularly evident at two periods in the prevalence survey; resolving the version compatibility problem which prevented storage of a biometric ID, and observing the difference in bypass rates between data collectors. The bypass option was introduced because survey staff reported frustrations trying to complete the registration process when they faced challenges such as failure to connect and risked falling behind on their recruitment targets. However, once the bypass option was available, a small number of survey staff used it routinely rather than attempting biometric registration of participants. Enhanced training and spare equipment may be advisable to prevent some of the observed problems in the field.

In the survey, men were more likely to refuse fingerprinting than women, and refusal was more common in Harare than Bulawayo or Mashonaland East. Refusal was most common in those with either less than primary education or postgraduate education, and in the lowest and highest socioeconomic quintiles. This bimodal distribution suggests two separate mechanisms may lead to refusal. However, because of its large sample size the study is highly powered to detect small differences between groups, and some observed effects may be due to chance.

We demonstrate the applicability of fingerprints to understand intervention coverage. As shown in other studies, females were more likely to use CHIEDZA services, and those who were not sexually active would have found these services less relevant to them.^12 13^ We observed much lower population-level coverage of the intervention than anticipated, combined with high mobility and association of length of residence with intervention access. Out-migration from the clusters may explain the discrepancy between the large number of CHIEDZA clients, as a proportion of residents and the low intervention coverage among survey participants. We found a strong relationship between distance and uptake, even in small clusters. The greater the distance from the hall, the lower the probability of awareness or utilisation of the service, consistent with the well documented inverse relationship between healthcare utilisation and distance.^14 15^ Nearly two-thirds of survey participants did not know that community-based youth-friendly SRH services existed in their area and, even among those who knew about their existence, nearly half did not use them. This finding underscores the need for intensive sensitisation to increase utilisation and is supported by findings from other studies where utilisation was low due to lack of knowledge.^12^ Services were not advertised using radio, television and social media to reduce the risk of contamination by giving information about CHIEDZA services to those in control clusters.

Three quarters (75.0%) of CHIEDZA clients were female, yet service uptake in the survey participants was only 32% higher in females than males (25.3% of females and 19.1% of males accessed services), not three times higher. This may partly be attributable to the population being skewed female (10 741/17 682 survey participants were female). Women were also more mobile and hence had less exposure time to CHIEDZA; 29.7% of surveyed women (3191/10741) and 15.2% of men (1054/6940) had resided at their current address <12 months, while 49.3% of women (49.3%) and 68.3% of men had been resident >3 years.

Uptake results in the control arm were particularly sensitive to comparison score cutpoint. Most matches in the control arm had low comparison scores, so when the cutpoint was raised they were no longer categorised as matches. It may be more appropriate to use different cutpoints in the intervention and control arms, since prior knowledge that a match is more likely in the intervention arm was not taken into account for the probability assessment. However, such a step would require careful justification in a randomised controlled trial.

The strength of this study was that it was based on data from a large randomly selected sample, representative of youth from three provinces of Zimbabwe from both urban and peri-urban communities, increasing generalisability of the findings. A limitation was the lack of information around what caused the ‘scanner not working’ or ‘scanner not connecting to tablet’ errors which prevented biometric registration of a large number of survey participants. Data collectors were motivated to complete their work quickly which may have hampered biometric registration. Refusal introduced bias into the sample of participants with a biometric fingerprint, whereas the selected sample of 17 682 participants was representative of the population. All demographic variables were self-reported, and the question on length of residence did not allow for part-time or interrupted residence.

## Conclusions

Digital fingerprinting offers a highly feasible and effective approach to evaluate intervention delivery within cluster randomised trials. There are additional challenges to using this technology in house-to-house surveys. Possible steps to address them include carrying spare equipment, and moving biometric registration to the end of the questionnaire to prevent wasted time during connection.

## Supplementary material

10.1136/bmjopen-2025-107583 online supplemental file 1

10.1136/bmjopen-2025-107583 online supplemental file 2

10.1136/bmjopen-2025-107583 online supplemental file 3

10.1136/bmjopen-2025-107583 online supplemental file 4

## Data Availability

Data are available upon reasonable request.
