# Supplementary figures and images for: Effectiveness and acceptability of biometrics to evaluate intervention coverage and contamination in a cluster randomised trial of community-based sexual and reproductive health services for youth in Zimbabwe
Source: BMJ Open. 2026 Jul 15;16(7):e107583. doi: 10.1136/bmjopen-2025-107583 (PMC13374400; doi:10.1136/bmjopen-2025-107583)

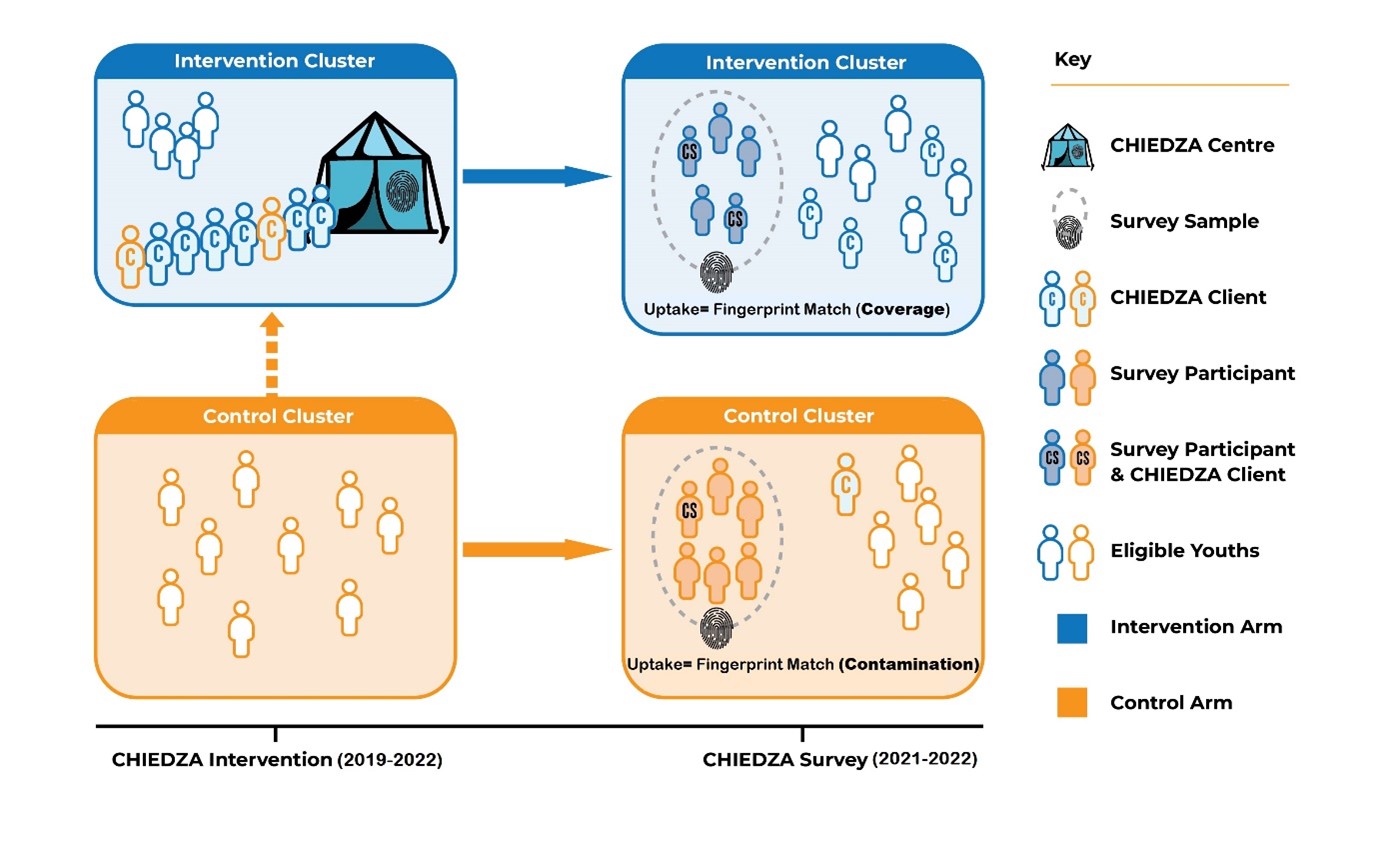

Supplement: online supplemental file 1 [file bmjopen-16-7-s001.jpg]

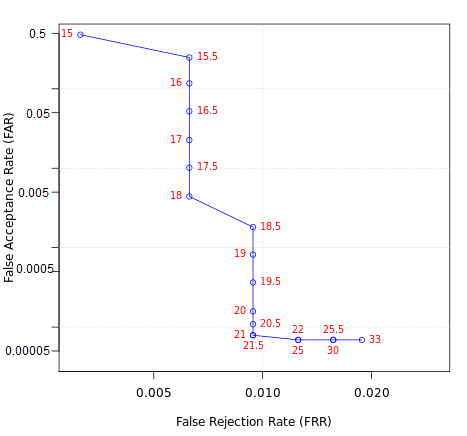

Supplement: online supplemental file 2 [file bmjopen-16-7-s002.png]

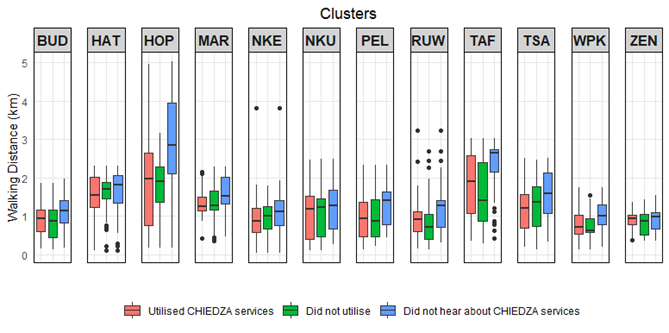

Supplement: online supplemental file 3 [file bmjopen-16-7-s003.png]
