# Supplementary material for: Effectiveness and acceptability of biometrics to evaluate intervention coverage and contamination in a cluster randomised trial of community-based sexual and reproductive health services for youth in Zimbabwe
Source: BMJ Open. 2026 Jul 15;16(7):e107583. doi: 10.1136/bmjopen-2025-107583 (PMC13374400; doi:10.1136/bmjopen-2025-107583)
Supplement: online supplemental file 4 [file bmjopen-16-7-s004.docx]

Table S1: Survey biometric registration outcomes by month

|  | October  2021 | November 2021 | December  2021 | January  2022 | February  2022 | March  2022 | April  2022 | May/June  2022 | Total |
| --- | --- | --- | --- | --- | --- | --- | --- | --- | --- |
| Completed biometric registration | 1817 | 2332 | 793 | 2370 | 1633 | 868 | 2128 | 1734 | 13675 |
| Bypassed registration | 1 | 30 | 24 | 18 | 22 | 18 | 306 | 816 | 1235 |
| Refused | 22 | 67 | 143 | 132 | 237 | 213 | 188 | 280 | 1182 |
| Compatibility issue | 243 | 0 | 0 | 0 | 0 | 0 | 0 | 0 | 243 |
| Scanner not working | 5 | 115 | 132 | 74 | 94 | 65 | 123 | 115 | 723 |
| Scanner did not connect to tablet | 9 | 80 | 66 | 70 | 69 | 48 | 43 | 117 | 502 |
| Low battery | 0 | 0 | 0 | 6 | 12 | 11 | 1 | 0 | 30 |
| Individual reasons | 1 | 21 | 24 | 2 | 1 | 1 | 2 | 1 | 53 |
| Not completed, unknown reason | 22 | 0 | 2 | 0 | 2 | 3 | 6 | 4 | 39 |
| Total | 2120 | 2645 | 1084 | 2672 | 2070 | 1227 | 2797 | 3067 | 17682 |

Table S2: service uptake by arm using different cutpoints

| Cutpoint | Uptake in intervention arm (N=6806) | Proportion of intervention arm matches that were in different clusters | Uptake in control arm (N=6869) |
| --- | --- | --- | --- |
| 21 | 1605 (23.6%) | 181 (11.3%) | 286 (4.2%) |
| 21.5 | 1574 (23.1%) | 161 (10.2%) | 252 (3.7%) |
| 22 | 1501 (22.1%) | 105 (7.0%) | 161 (2.3%) |
| 23 | 1451 (21.3%) | 64 (4.4%) | 87 (1.3%) |
| 24 | 1424 (20.9%) | 50 (3.5%) | 65 (1.0%) |
| 25 | 1410 (20.7%) | 48 (3.4%) | 60 (0.9%) |
