# Supplementary material for: Effectiveness and acceptability of biometrics to evaluate intervention coverage and contamination in a cluster randomised trial of community-based sexual and reproductive health services for youth in Zimbabwe
Source: BMJ Open. 2026 Jul 15;16(7):e107583. doi: 10.1136/bmjopen-2025-107583 (PMC13374400; doi:10.1136/bmjopen-2025-107583)
Supplement: Supplementary file 5 [file bmjopen-2025-107583.reviewer_comments.pdf]

PEER REVIEW HISTORY

BMJ Open publishes all reviews undertaken for accepted manuscripts. Reviewers are asked to complete a checklist review form and are provided with free text boxes to elaborate on their assessment. These free text comments are reproduced below.

This paper was submitted to another journal from BMJ but declined for publication following peer review. The authors addressed the reviewers' comments and submitted the revised paper to BMJ Open. The paper was subsequently accepted for publication at BMJ Open.

ARTICLE DETAILS

Title (Provisional)

Effectiveness and acceptability of biometrics to evaluate intervention coverage and contamination in a cluster randomised trial of community based sexual and reproductive health services for youth in Zimbabwe

Authors

Simms, Victoria; Bandason , Tsitsi; Dauya, Ethel; Dziva Chikwari, Chido; Grundy, Chris; Tembo, Mandikudza; Mavodza, Constancia; Mugurungi, Owen; Apollo, Tsitsi; Ashrafee, Riffat; Demetriou, Demetris; Sharma, Anindya; Hayes, Richard; Kranzer, Katharina; Ferrand, Rashida A.

VERSION 1 - REVIEW

**Reviewer**	**1**
**Name**	**Reviewer 1**
**Affiliation**	
**Date**	**10-Jun-2025**
**COI**	

Comments not published pending reviewer's consent.

VERSION 2 - REVIEW

**Reviewer**	**2**
**Name**	**Woodworth, Simon**
**Affiliation**	**University College Cork National University of Ireland, Business Information Systems**
**Date**	**17-Nov-2025**

**COI**	**None**

Line 95: Correct "feaisble"

The paper addresses a perennial issue of identification with studies in sub Saharan Africa. The results are broadly applicable, even to studies of substantially different design.

The reviewer's experience has shown that extensive training is needed in the operation of tablets and connected equipment. While authors will appreciate this evidence is somewhat anecdotal, they may wish to consider enhanced training as well as more equipment to mitigate the stated problems in the field.

**Reviewer**	**3**
**Name**	**Kelson, Zoe**
**Affiliation**	**University of Exeter, Mathematics**
**Date**	**21-Apr-2026**
**COI**	**None**

This study investigated the feasibility of digital fingerprints to assess intervention coverage and contamination in a CRT of community-based sexual and reproductive health services for youth (CHIEDZA).

Reviewer comments:

"A population based survey of youth aged 18-24 (700/cluster) was conducted to ascertain impact"

and

"Between April 2019-March 2022, 36,9991 clients attended the CHIEDZA service and 99.9% used biometric registration. In the survey 13,675/17,682 (77.3%) participants completed biometric registration: 1182 refused, 1235 bypassed and 1590 could not register. "

and

"If the sample was inadequate to recruit 700 youths, a further sample of road sections was taken and visited sequentially to maintain randomness until 700 was reached. A median 100 sections (IQR 88-148) were surveyed per cluster. All those eligible were contacted, if possible, and asked to participate in the survey and provide fingerprint registration"

and

"In total 17,682 participants were enrolled of whom 13,675 (77.3%) successfully registered their fingerprint (Figure 1). Reasons for non-registration were that the researcher bypassed the process (N=1235), the participant refused to give fingerprints (N=975), or the registration process was begun but not completed (N=1797). Of those who started the

process but did not complete it, 207 refused (95 for religious reasons, 66 because of data concerns, and 46 gave no reason), for a total of 1182 refusals (975+207). "

Can the authors please further comment on whether the included cohort can be considered to be representative?

"The CRT primary outcome was prevalence of unsuppressed HIV viral load among youth living with HIV, evaluated using a cross-sectional population-based survey of 700 youth aged 18-24 years per cluster."

Can the authors please communicate an assessment of sample size for this study, suitably accounting for the clustered design?

"variables collected included..."

Can the authors please confirm if there was any missing data?

"Chi-square tests were used to investigate whether demographic characteristics were associated with refusal of biometric registration"

A suitable statistical test has been applied by the authors.

Did the authors consider adjusting this analysis for cluster?

"Multilevel logistic regression estimated the association of hall distance with service uptake."

and

"Multi-level mixed-effects logistic regression was used to examine the association of service utilisation with walking distance and other covariates, accounting for cluster"

Appropriate modelling methods have been conducted by the authors, satisfactorily accounting for clustering and potential confounding in the data.

"Table 1: Survey biometric registration refusal by sociodemographic and trial characteristics, among those who did not bypass"

and

"Table 2:Service uptake among prevalence survey participants who completed biometric registration (N=13675); using cutpoint 21.5)"

and

"Table 3: Utilisation of CHIEDZA services in intervention arm by sociodemographic characteristics"

Can race and ethnicity please be included in the study?

"Figure S3: Mean walking distance to CHIEDZA service location by uptake of service"

Can groups under comparion in this analysis please be statistically tested for significant differences?

"A limitation was the lack of information around what caused the ‘scanner not working’ or ‘scanner not connecting to tablet’ errors which prevented biometric registration of a large number of survey participants"

Can the discussion on the study limitations please be expanded on by the authors?

Thanks for providing a copy of the STROBE checklist.

VERSION 2 - AUTHOR RESPONSE

Reviewer: 2

1. Line 95: Correct "feaisble"

Done.

2. The paper addresses a perennial issue of identification with studies in sub Saharan Africa. The results are broadly applicable, even to studies of substantially different design. The reviewer's experience has shown that extensive training is needed in the operation of tablets and connected equipment. While authors will appreciate this evidence is somewhat anecdotal, they may wish to consider enhanced training as well as more equipment to mitigate the stated problems in the field.

We have added this suggestion to the recommendations.

Reviewer: 3

This study investigated the feasibility of digital fingerprints to assess intervention coverage and contamination in a CRT of community-based sexual and reproductive health services for youth (CHIEDZA).

Reviewer comments:

1. "A population based survey of youth aged 18-24 (700/cluster) was conducted to ascertain impact"
   and
   "Between April 2019-March 2022, 36,9991 clients attended the CHIEDZA service and 99.9% used biometric registration. In the survey 13,675/17,682 (77.3%) participants completed biometric registration: 1182 refused, 1235 bypassed and 1590 could not register. "
   and
   "If the sample was inadequate to recruit 700 youths, a further sample of road sections was taken and visited sequentially to maintain randomness until 700 was reached. A median 100 sections (IQR 88-148) were surveyed per cluster. All those eligible were contacted, if possible, and asked to participate in the survey and provide fingerprint registration"
   and
   "In total 17,682 participants were enrolled of whom 13,675 (77.3%) successfully registered their fingerprint (Figure 1). Reasons for non-registration were that the researcher bypassed the process (N=1235), the participant refused to give fingerprints

(N=975), or the registration process was begun but not completed (N=1797). Of those who started the process but did not complete it, 207 refused (95 for religious reasons, 66 because of data concerns, and 46 gave no reason), for a total of 1182 refusals (975+207). "
Can the authors please further comment on whether the included cohort can be considered to be representative?

The originally selected sample was representative, but the subgroup with a registered fingerprint had limited representativeness due to the association of refusal or failure with demographic and cluster-related factors. We have added this to the paper.

2. Can the authors please communicate an assessment of sample size for this study, suitably accounting for the clustered design?

The sample size calculation is shown in the survey protocol paper which is referenced (reference 10). It is based on the expected prevalence of HIV and the expected effect of the CHIEDZA intervention on trial outcomes. Including it in this paper would require some background explanation about the CHIEDZA trial, which would take up space and is not relevant to the aim of this paper. Therefore we decided not to show it here.

3. Can the authors please confirm if there was any missing data?

Sex was missing for one participant. Details of missing data have been added to Table 1.

4. "Chi-square tests were used to investigate whether demographic characteristics were associated with refusal of biometric registration". A suitable statistical test has been applied by the authors. Did the authors consider adjusting this analysis for cluster?

We have replaced the chi square tests with univariable mixed-effect logistic regression models adjusting for cluster. We made an exception for trial arm, since it is a cluster-level variable. We have added an explanation of this method to the Data Analysis section of the methods, and updated the results in Table 1.

5. Appropriate modelling methods have been conducted by the authors, satisfactorily accounting for clustering and potential confounding in the data.

Thank you.

6. Can race and ethnicity please be included in the study?

Race and ethnicity data were not collected. We believe all participants were black Zimbabweans and the great majority (>99%) were of Shona ethnicity.

7. "Figure S3: Mean walking distance to CHIEDZA service location by uptake of service". Can groups under comparion in this analysis please be statistically tested for significant differences?

The statistical analysis is presented in Table 3.

8. "A limitation was the lack of information around what caused the ‘scanner not working’ or ‘scanner not connecting to tablet’ errors which prevented biometric registration of a large number of survey participants". Can the discussion on the study limitations please be expanded on by the authors?

This discussion has been expanded in the final paragraph before the conclusion.
